# PHND: Pashtu Handwritten Numerals Database and deep learning benchmark

**DOI:** 10.1371/journal.pone.0238423

**Published:** 2020-09-02

**Authors:** Khalil Khan, Byeong-hee Roh, Jehad Ali, Rehan Ullah Khan, Irfan Uddin, Saqlain Hassan, Rabia Riaz, Nasir Ahmad

**Affiliations:** 1 Department of Electrical Engineering, University of Azad Jammu and Kashmir, Muzaffarabad, Pakistan; 2 Department of Computer Engineering, Ajou University, Suwon, South Korea; 3 Department of Information Technology, College of Computer, Qassim University, Al-Mulaida, Saudi Arabia; 4 Department of Information Technology, Superior University, Lahore, Pakistan; 5 Institute of Culture and Society, University of Navarra, Pamplona, Spain; 6 Department of Computer Science, University of Azad Jammu and Kashmir, Jammu and Kashmir, Muzaffarabad, Pakistan; 7 Department of Computer Systems Engineering, University of Engineering and Technology, Peshawar, Pakistan; Vietnam National University, VIET NAM

## Abstract

In this paper we introduce a real Pashtu handwritten numerals dataset (PHND) having 50,000 scanned images and make publicly available for research and scientific use. Although more than fifty million people in the world use this language for written and oral communication, no significant efforts are devoted to the Pashtu Optical Character Recognition (POCR). We present a new approach for Pahstu handwritten numerals recognition (PHNR) based on deep neural networks. We train Convolutional Neural Networks (CNNs) and Recurrent Neural Networks (RNNs) on high-frequency numerals for feature extraction and classification. We evaluated the performance of the proposed algorithm on the newly introduced Pashtu handwritten numerals database PHND and Bangla language number database CMATERDB 3.1.1. We obtained best recognition rate of 98.00% and 98.64% on PHND and CMATERDB 3.1.1. respectively.

## Introduction

The emergence of information technology and extensive use of off-line and on-line digital text are increasing the need for conversion of newspapers, novels, newspapers, and old manuscripts into computer-readable form. Optical Character Recognition (OCR) is a system that converts handwritten and machine-written scanned images into machine-editable form. It is one of the most researched areas in pattern recognition, which significantly matured in the last 20 years [1–3]. Sources of the text images are scanned/camera-based documents, text images from scenes, and video captions of broad-casted videos. The OCR system was researched for the first time around 65 years ago [4]. Since then, endeavors have been done by researchers, ultimately leading to a mature OCR system for most of the languages of the world. OCR systems have been designed for this purpose for different languages of the world like English, German, French, Chinese, Korean, etc. Despite these large scale developments, the OCR system for cursive scripts languages (Arabic, Pashtu, Urdu, etc.) is still a challenging job.

Pashtu is a language spoken by 50 million people in the world [5]. It is the national language of Afghanistan and also spoken in the two largest provinces of Pakistan, including Khyber Pakhtunkhwa and Baluchistan. It is a language written in a complex way by calligraphers. Pashtu language is associated with very rich literature and heritage. There is enormous written material, addressing diverse areas such as politics, religion, education, culture, poetry, and music. Cursive script languages that share some characteristics with Pashtu are Arabic, Urdu, and Persian. OCR systems for these languages exit at some stages, for example [6–8]. However, due to less research work regarding the Pashtu text recognition, this language still requires a mature OCR system.

Significant research has been done in the field of OCR for different languages of the world, but unfortunately, POCR is still far away due to certain major reasons. Unlike many other languages, Pashtu is a cursive language which is written from the right side to left. Languages that are most similar to Pashtu are Arabic, Persian, Urdu. Non-cursive script languages such as English, French etc. are inherently easy as for as machine-based recognition is concerned, as very little variation occurs in individual character shape. However, the cursive script languages such as Pashtu, Arabic, and Urdu have very complicated word formation rules [9]. Some of these complexities include space omission and space insertion due to separate letters [10], variations of individual characters, complexity in isolated character segmentation, baseline detection, etc. Furthermore, in these cursive scripts languages, when individual characters join, an intermediate shape is formed called ligatures. These ligatures are missing in non-cursive script languages. Due to such complexities, recognition of the Pashtu language is far away from maturity level.

Our current research work is part of a long term research strategy, ultimately leading to a mature OCR system for Pashtu. In this regard, as an initial step, we develop the first Pashtu handwritten numerals dataset, i.e., PHND. We develop a CNNs based recognition system for Pashtu numerals.

To summarize, contributions of this paper are as follows:

- We propose a new dataset PHND for handwritten Pashtu numerals. The dataset consists of 50, 000 scanned images of both male and female candidates.
- We build CNNs and RNNs based numerals recognition models for cursive script languages using Pashtu language numerals as a case study.
- We also assess the performance of the CNNs based model on the Bangla digits database and obtaining competitive results.

## Related work

Literature reports that two prominent methods were previously used for the OCR system of cursive script languages [11], including analytical and holistic approaches. In the following paragraphs, these methods are briefly discussed. A discussion is also presented how POCR was previously addressed through different methods.

Analytical methods are advanced level modeling methods which are based on certain grammatical rules for the respective language and script. The shape of each character is identified by a unique set of features such as loop, hole, etc. These algorithms need segmentation at the atomic level for better performance, which is itself another challenge. These methods work well in cases where segmentation at the atomic level is comparatively easy. For example, in English language text, each character boundary can easily be located through some set of algorithms. In a nutshell, to achieve acceptable performance, we need nearly perfect segmentation at the atomic level in analytical methods.

Neural Networks (NNs) and Hidden Markov Models (HMMs) are frequently used [12, 13] for cursive script languages OCR systems. Both of these systems were tested on the Arabic language. The recognition rate obtained was 93% on 138 pages of the Arabic language. Some other methods which addressed the OCR system for cursive script languages through analytical methods can be found in ICDAR [14, 15].

Th second class of methods is holistic methods that follow no typography rules. These are very generic methods and can be applied to any text and any language. An image having an isolated word or ligature is considered a one-dimensional feature vector, and some features are extracted from it. There is no need for segmentation at the atomic level. The main drawback of these methods is a large amount of ligature data is needed for training and testing. Most of these methods are robust to location, scale, and rotation changes. Moreover, these methods need are a rich set of features extraction for discrimination of different classes. The extracted features must be invariant to location, orientation, and scaling. These features must be reliable under noise conditions as well.

One of the popular holistic based OCR systems is BBN Byblos OCR [16]. This method has been tested on multiple languages of the world. The method was evaluated on different languages as well as many scripts of the same language. The error rate reported was quite low for synthetic data. However, very few ligatures were included in the training and validation sessions. These methods fail to perform when applied to larger databases having much variety in character and ligature sets.

A multi-tier holistic approach for Pashtu text was developed in [17]. The authors evaluated their work with very little training and testing data. The authors of the paper tested their algorithm for the Noori Nastaliq script. As compared to Naksh script, Nastaliq is more complicated as it is obtained through calligraphy and is more complex to recognize. The system was tested on synthetic data, and final recognition was performed through a feed-forward backpropagation neural network. A method proposed in [18] is also using a holistic approach. The authors of the paper claimed the method robust to changes in scale, rotation, and location. The research proposed in [18] was considered as a test case for cursive script languages and initially applied to Pashtu. The authors also introduced a database of 8, 000 images having 1, 000 ligatures. The proposed framework was based on a scale-invariant feature transform along with segmentation. As mentioned earlier, there is no significant research work for POCR systems; some early research work addressing POCR is listed in the references [19–23].

Recently introduced Convolutional Neural Networks (CNNs) showed excellent recognition performance in different visual recognition tasks, including OCR systems [24]. A method proposed in [24] is for Pashtu text particularly. The authors of the paper introduced the first text image database for handwritten ligatures called KPTI. The KPTI consists of 17, 015 images of Pashtu text taken from different handwritten Pashtu books. A deep learning-based method through Bi-direction and Multi-Dimensional long and short term memory networks were applied to the KPTI database. To the best of our knowledge, this is the latest work on the POCR system thus far.

Deep learning-based methods are already applied to the isolated characters and numerals of different languages [25–28]. Our current research work has two main contributions. Firstly, we introduce a new Pashtu numerals database PHND. We applied two of these deep learning method called CNNs and RNNs to PHND. We also tested the CNNs based model on another cursive script numeral database called CMATERDB 3.1.1.

**Fig 1:**
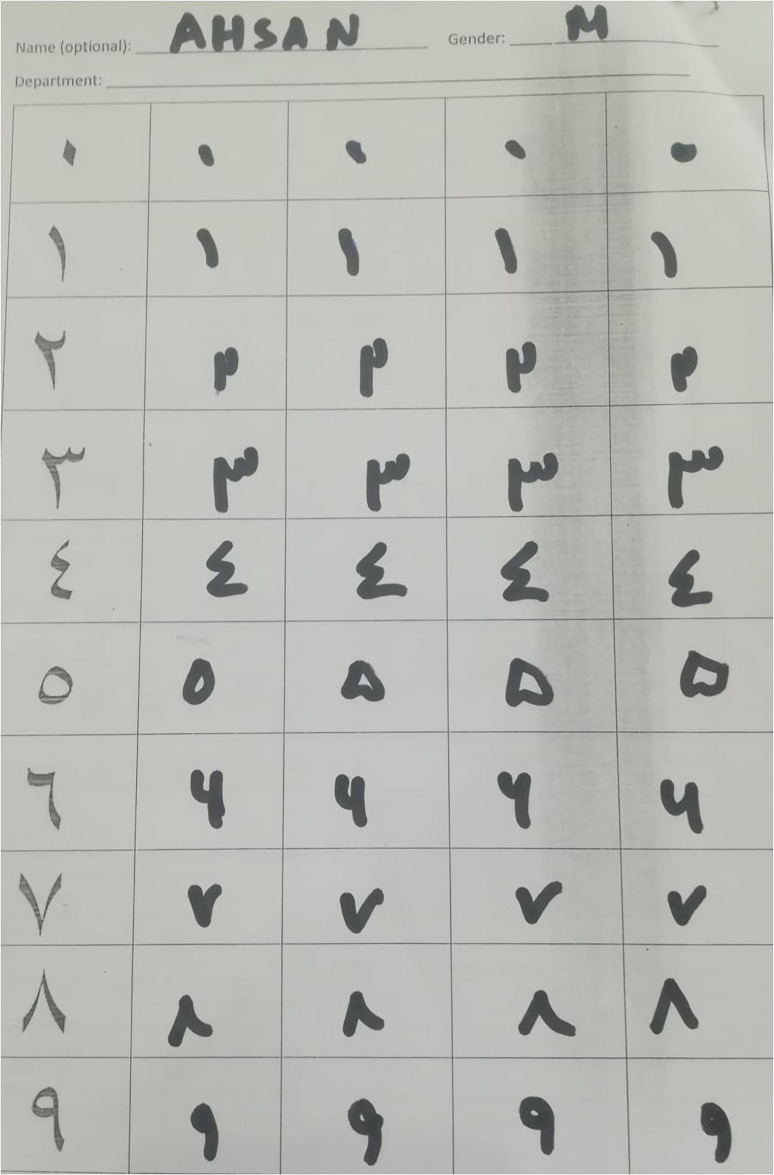
PHND form filled by a participant which was distributed among faculty members and students of three different universities.

Fig 1 shows a form filled by a participant for distribution among faculty members and students. The individual in this manuscript has given written informed consent (as outlined in PLOS consent form) to publish these case details.

## Pashtu Handwritten Numerals Database (PHND)

A critical disadvantage of deep learning-based methods reported by researchers is the requirement of a large amount of training data. One of the main contributions of this paper is introducing a new database; we called PHND. This database is freely available for research purposes https://data.mendeley.com/datasets/xv3kdy7r6k/2. To the best of our knowledge, this is the first database ever introduced for Pashtu handwritten numerals.

**Fig 2:**
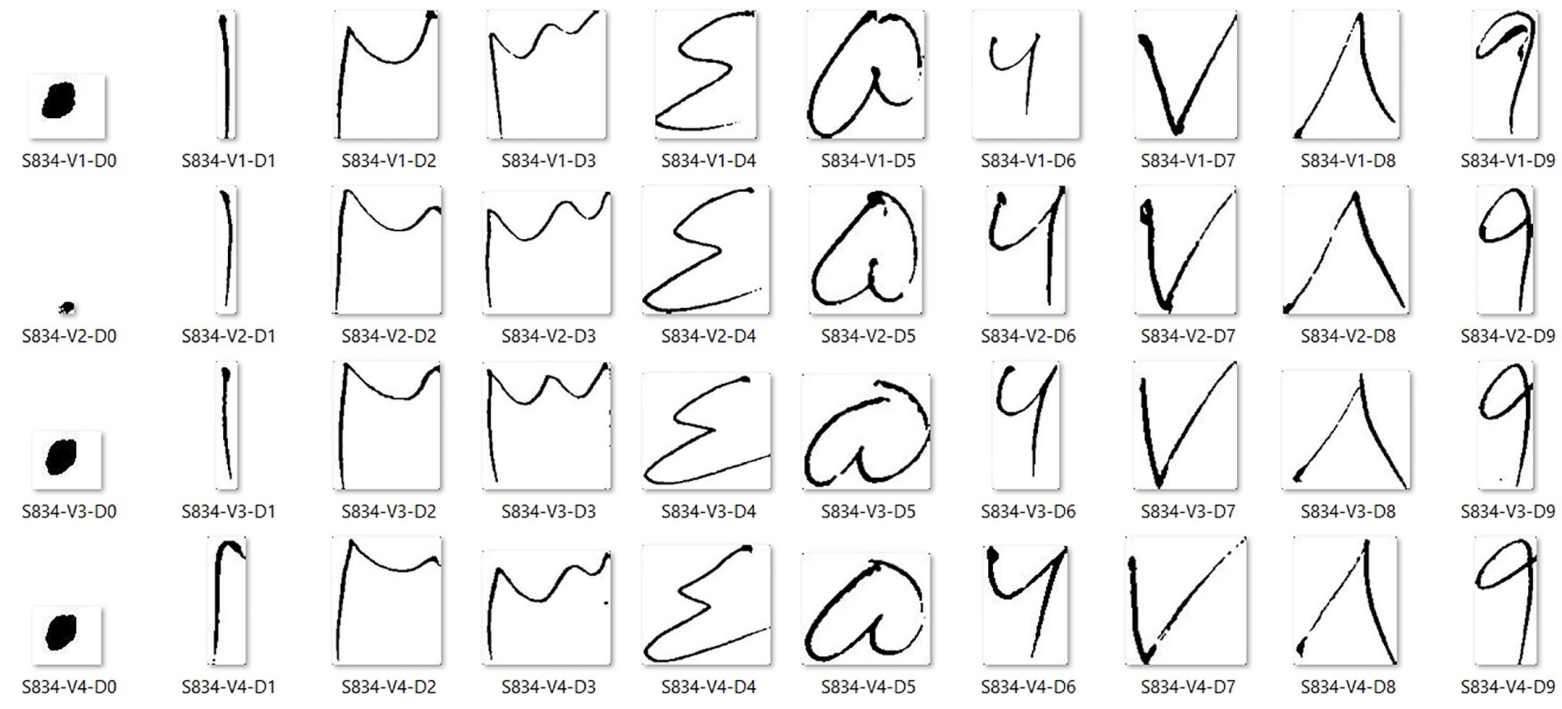
Images in the folder, with name format.

To ensure diversity in writing style among candidates, data was collected from three different regions of the country. All numerals in the PHND are handwritten images collected from faculty members and students of three various universities of Pakistan including, the University of Azad Jammu and Kashmir, University of Malakand, and the University of Peshawar. A total number of 1250 (male: 65% and female: 35%) candidates participated in writing the text. All the individuals has given written informed consent (as outlined in PLOS consent form) to publish these case details. Each subject wrote each digit four times (0-9). All the images were scanned as greyscale images, which were later on converted into binary form. Fig 2 shows a form distributed among faculty members and students for writing. Fig 2 shows a form filled by a participant for distribution among faculty members and students. The individual in this manuscript has given written informed consent (as outlined in PLOS consent form) to publish these case details. Each digit in the form is written four times. A single sheet per person contributes 40 characters. To bring more diversity in the database, no subject was repeated for writing the text again.

All candidates in the database were in the age range of 18-60 years. After writing, each page was scanned with a 300 DPI resolution. Before converting images to binary form, some preliminary preprocessing steps were performed listed below:

- The inclination of every page was corrected with a horizontal histogram.
- The center of each digit was detected first. In the proposed work, we use the connected component algorithm for localizing center of each digit.
- Every digit was extracted from the image and then rescaled to a fixed size 80 × 80.
- After performing all the above steps, images were converted into binary form. Some images after performing these pre-processing steps are shown in Fig 2.

All images in the PHND are arranged in a specific order. An example folder with all 40 images with corresponding labels is shown in Fig 2. Each image name has three parts, i.e., S, V, and D. The English alphabet S shows the subject number, which varies from 1-1250. The alphabet V shows a version of writing since each subject wrote each digit four times, so it is in the range 0-3. And lastly, the alphabet D shows digit number, which varies from 0-9.

So far, we collected these handwritten forms from 1250 participants comprised of women and men from faculty members and students of the universities, as mentioned above. We took an interest in keeping record of participant’s age, gender, and if they are left-handed or right-handed. As this research is part of our long-term research strategy. Even though this information (age, gender, etc.) had no significance at this stage, it could be used in the future while developing a mature POCR and its later applications.

## Deep learning

In the last few years, deep learning methods (DLMs) have shown excellent performance in various areas of pattern recognition and machine learning. Deep Neural Networks (DNNs) include Deep Belief Networks (DBNs), CNNs, and Stacked Auto Encoder. As compared to shallow learning approaches [29], DNNs are more efficiently representing highly non-linear function. The most probable reason for the mentioned fact is the composition of many layers. The DLM usually takes raw images as inputs without extracting features. The first two levels (low and middle-level layers) of DNNs extract features and the final level performs the classification task. The final layer of the DNNs uses feed-forward neural networks (FFNs). The DNNs is a structured framework which is integrated with all necessary modules within a single unified framework. The DNNs based models reported much better accuracy as compared to state-of-the-art (SOA) methods [30–37].

In the Multilayer Back-propagation (MBP) algorithm, the final classification layer propagates the error signal in backward direction through each layer. Based on the output layer error, the connection weights are updated regularly. The performance of the MBP algorithm is poor when the number of hidden layers is too much. This condition is called Diminishing Gradient Problem (DGP) [25, 29]. The DGP occurs as the error signal becomes too smaller, eventually updating of the weights in the first layers is stopped.

A new approach was proposed in [38], which is based on greedy layer-wise training. This new approach overcomes the DGP problem and is as known as DBNs. In the DBNs, the classification error is minimized by first pre-training the weights through an un-supervised training method from the last (bottom most) layer. Then fine-tuning of the weights is performed through supervised learning methods [39]. This work provided a breakthrough to the researchers working on DLMs. Moreover, the Restricted Boltzmann Machines (RBMs) provided a way to update the un-supervised learning part [40].

## Convolutional neural networks

In recent days CNNs have shown remarkable results in different visual recognition tasks. The CNNs structure was proposed initially in 1980 by Fukushima [41]. Due to certain drawbacks such as requirements of large datasets and high computational cost, CNNs did not gain much attention in the early 1990s [37]. After that, the CNNs based methods are further improved, and better results are reported in various pattern recognition tasks. Recently, multi-column CNNs methods are applied for alpha-numerals, digits, traffic signals, and some other objects classification [42–44]. The CNNs based methods reported outstanding results compared to convention records on several benchmark datasets, including MNIST [45] digits dataset and CIFAR-10 [46]. As compared to other DNNs methods, CNNs have some extra properties, for example, the CNNs designed is based on the human visual processing system, and its structure is more optimized to 2D features. A pooling layer very effectively extracts the shape variations. The CNNs architecture composed of sparse connections with tied weighting elements that need fewer parameters compared to a fully connected network having a similar size. The CNNs suffers less from DGP as its training strategy is based on gradient-based learning (GBL) methods.

The advent of fast graphical processing units and the development of much bigger datasets such as Image Net [47] significantly reduce the computation cost and enhance the performance of different learning tasks in computer vision. Consequently, CNNs are the most popular and efficient method for different visual recognition tasks [48]. In the last few years, CNNs have been applied to various pattern recognition problems, including OCR [49], object detection [50], face recognition [51] and much more.

## Proposed method

In this Section we present details of the proposed method. Our proposed method uses an appearance-based technique, in which image is considered as a one-dimensional vector, and some features are extracted from it. For feature extraction, we use CNNs and then perform classification with Soft-Max.

### Architecture

**Fig 3:**
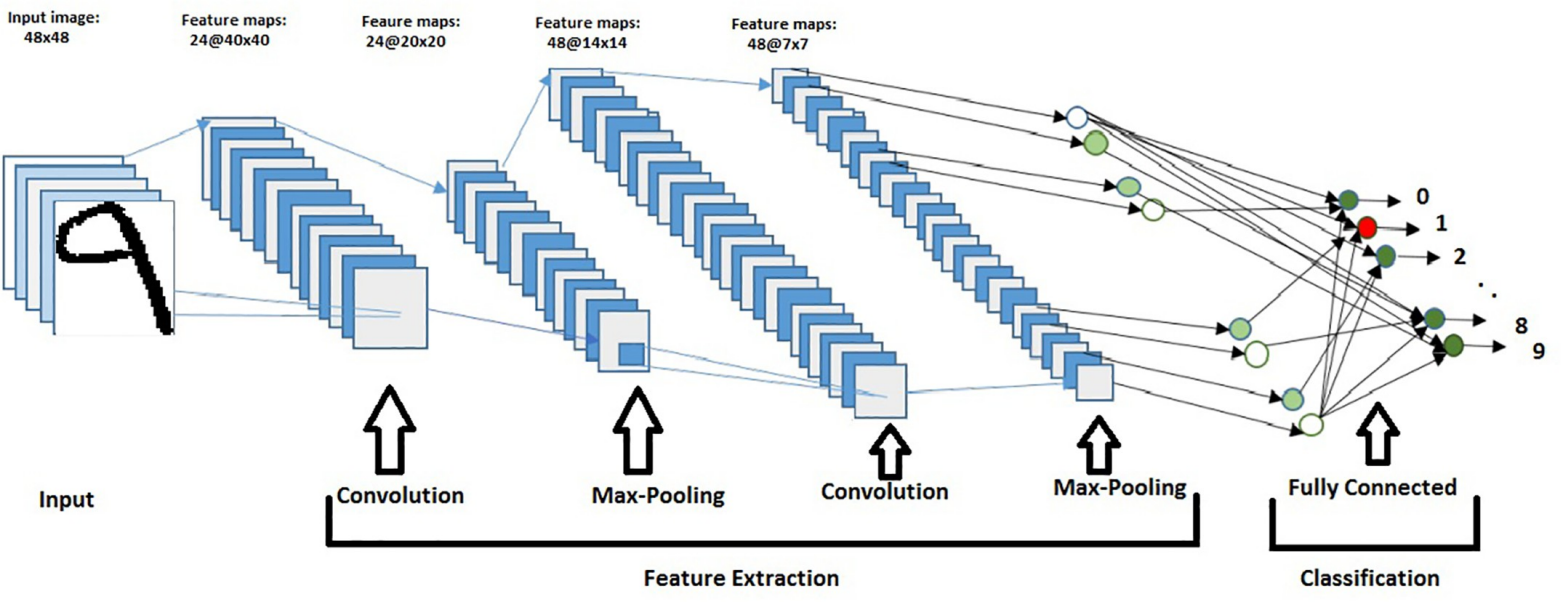
Proposed CNNs architecture.

There are several parameters in CNNs which significantly affect the performance of the CNNs based model. For example, the kernel sizes, the number of convolutional layers, and the number of filters in each convolutional layer. The CNNs architecture we used is shown in Fig 3. We used two convolutional layers with 24 (9 × 9) and 48 (7 × 7) filters, respectively. In each layer, we used the rectified Linear Unit (Re-Lu) as an activation function. Each convolutional layer is followed by the Max-Pooling layer.

The input data *y*_1_, *y*_2_ to the CNNs are images. Formally, the input image to the first convolutional layer is images represented as *M* × *N* × *C*, where M is the image height, N width, and C color channel.

A CNNs framework consists of convolutional layers (CLs), Max-Pooling layers (MPLs), and fully connected layer (FCL). The CLs will have a number of filters or kernels represented as *N* × *M* × *C*, M, and N representing height and width of the kernel and C channel. We obtained the feature map by convolving kernel with image contents. The MPLs will have kernels or filters of size *P* × *Q*, where *P* is representing height and *Q* width of the MPL. FCL follows a set of CLs and MPLs. For the proposed work, all these layers are presented in Fig 3.

### Layers

#### Convolutional layers

The CLs typically extract certain features from images such as edges, edge points, corners, etc. We convolved *P* × *Q* filter with image contents through a stride of 1 pixel. Let the first part of the CLs is represented with *l* then input to the next set of CNs layer *l*-1 is computed as:

$$\begin{array}{c} X_{i}^{l}=A_{i}^{l}+\sum_{b=1}^{M}\sum_{c=1}^{M}W_{i}Y_{\left(i+b,j+c\right)}^{l-1} \end{array}$$ (1)

Where the bias matrix is represented by $A_{i}^{l}$ and the filter or kernel moving across the image by *W*_*i*_. We applied the activation function to the CLs which is represented as;

$$\begin{array}{c} Y_{i}^{l}=\sigma \left(X_{i}^{l}\right) \end{array}$$ (2)

We used Rectified Linear Unit (Re-Lu) as an activation function in the proposed work.

$$\begin{array}{c} \sigma \left(X_{i}^{l}\right)=max\left(0,X_{i}^{l}\right) \end{array}$$ (3)

We used Re-Lu [52] as activation function, as it helps to increase the non-linear properties for the decision function as well as the overall network, as it effects immensely the respective field of the CNs.

#### Pooling layers

The pooling layer takes small patches from the CLs output, down-samples it, and produces a single output. The precision of the translation effect is reduced by reducing the resolution of the image. For pooling layers, various concepts exist in the literature, such as Max pooling, Min Pooling, Average Pooling, etc. We used Max pooling in our research work, which takes the maximum value of the block, which is under pooling. We used a 2 × 2 pixel window in our work.

#### Fully connected layers

After a set of CLs and MPLs the final content is fed to FCL. Images are flattened and then fed to the FCL. An FCL takes all neurons from the previous layer and connects it to every single neuron it has. Fig 3 shows the whole architecture that how various layers are connected with each other.

The classification task was performed in FCL. Image size was kept 80 × 80 in the whole framework. Details of the CNNs architecture is summarized in Table 1.

**Table 1:** CNNs parameters setting for the training process.

Parameters	vales
Batch size	128
Epochs	30
Momentum	0.9
Base learning rate	10^−4^

Similarly, each convolutional layer information is summarized in Table 2

**Table 2:** CNNs layers information.

Layer	Filter size	output size
Input	–	48 × 48 × 1
1st Convolution	9 × 9	40 × 40 × 24
1st Max-Pooling	2 × 2	20 × 20 × 24
2nd Convolution	7 × 7	14 × 14 × 48
2nd Max-Pooling	2 × 2	7 × 7 × 48
FCL layer (Soft-Max)	–	number of classes (10)

### CNNs optimization

#### Learning rate

The weight update of the network is done through the learning rate represented as *α*. The learning rate determines the convergence and generalization of the CNNs. The convergence of the network will be slow if the value of *α* is small, and divergence will occur if *α* is sufficiently large.

#### Activation function

We used the Re-Lu as the activation function in our work. To the output of each CLs and FCL, Re-Lu was applied. The Re-Lu helps in increasing the non-linear properties of the decision function as well as the overall network without affecting receptive fields of the CLs. This activation function maps more plausibly to neurons, improves the generalization ability of the network, and reduces the computation cost of the deep learning model.

#### Gradient descent

Both statistical estimation and machine learning consider the problem of minimizing an error function. This error function is represented as;

$$\begin{array}{c} Q\left(\omega \right)=\frac{1}{\alpha }\sum_{i=1}^{\alpha }Q_{i}\left(\omega \right) \end{array}$$ (4)

The iteration number is represented by i in Eq 4, the learning rate *α* > 0, and the parameter vector by Q. Where the parameter *ω* that minimizes *Q*(*ω*) is to be estimated.

When used to minimize the error function, a standard gradient descent method would perform the iterations as:

$$\begin{array}{c} \omega ≔\omega -\alpha \nabla Q(\omega ) \end{array}$$ (5)

#### Mini-batch

The gradient descent evaluates the gradient and updates all the parameters through a subset of training data. We called this subset of data as mini-batch. In mini-batch optimization, the entire dataset is divided into small batches, computing the gradient descent for a single batch, making the update, and moving to the next batch. The gradient uses the mini-batch as an iteration for each evaluation. The loss function is minimized with every single iteration. The full pass of the entire training data using mini-batch is called Epoch. We fixed the mini-batch size 125 and the number of epochs 30.

#### Momentum

It is most probable that gradient descent algorithm will oscillate towards the steepest path while moving to optimum. To prevent this oscillation, we added a momentum term to the parameters update. The updating will be:

$$\begin{array}{c} Q_{i+1}=Q_{i}-\alpha \nabla E\left(Q_{i}\right)+\gamma \left(Q_{i}-Q_{i-1}\right) \end{array}$$ (6)

Where the symbol *γ* determines how the previous gradient step contributed to the present iteration, the input data shuffles by default in this process.

#### Regularization

Over-fitting is a normal problem occurring in the training phase of machine learning methods. We added a regularization term for the weights to the loss function represented as *EQ*_*i*_. After adding the regularization term to the loss function, the loss function takes the form:

$$\begin{array}{c} E_{R}Q_{i}=EQ_{i}+\lambda \Omega \left(\omega \right) \end{array}$$ (7)

In Eq 7 *ω* represents the weight factor, λ is the coefficient added for regularization, and lastly Ω(*ω*) is the regularization function which is:

$$\begin{array}{c} \Omega \left(\omega \right)=\frac{1}{2}\omega^{t}\omega \end{array}$$ (8)

#### Soft-max classifier

The soft-max classifier shown excellent performance in different multi-class classification methods. It is particularly helpful in probabilistic classification strategies. For obtaining probability value for different classes, we applied Soft-max to the output units of the network:

$$\begin{array}{c} softmax\left(y_{i}\right)=\frac{e_{i}^{y}}{\sum_{k=1}^{N}e_{k}^{y}} \end{array}$$ (9)

#### Network parameters

To assess the complexity of the proposed architecture, the number of network parameters plays a key role. Comparison between different architecture can be made through these parameters. The dimension of the output feature map can be expressed and computed as:

$$\begin{array}{c} D_{f}=\frac{I-F}{S_{stride}}+1 \end{array}$$ (10)

where *D*_*f*_ refers the dimension of the feature map fed to FCL, I denotes the input feature maps, F is the filter to be convolved with I and *S*_*stride*_ represents stride in the convolution process.

A single layer parameters are obtained through:

$$\begin{array}{c} P_{i}=\left(F\times F\times Fmap_{i-1}\right)\times Fmap_{l} \end{array}$$ (11)

where *P*_*i*_ refers the total parameters in the *i*_*th*_ layer, *Fmap*_*l*_ is the output feature maps for *i*_*th*_ layers, and *Fmaps*_*i*−1_ is the total number of feature maps in the (*i* − 1)_*th*_ layer.

## Recurrent neural network

Along with CNNs, we also performed experiments using Recurrent Neural Network (RNNs). The MLPs map input vector into output without considering the previous computations at output unit [53, 54], whereas RNNs had much flexibility of tracking back previous computations. In this way, the previous history takes some part in computations at hidden layers. Similarly, the internal state of network is retained, which makes sufficient influence at the output level. RNNs use their feedback connection, which exists in the specific hidden layer to retain recent calculations contributing in the weight calculation of the current node in a particular sequence. When the input to the system is complex, in such cases, the time lag for retaining computation is difficult to maintain. Consequently, we can lose important information. In this situation, the information needed for a longer period of time are vanished from the cell’s memory. For addressing this special problem, a new concept of long short term memory (LSTM) networks was introduced [53].

The hidden layer memory cells are replaced in LSTM with memory blocks by using additional multiplicative units. These particular multiplicative units are mainly responsible for keeping information for a comparatively longer time. In certain conditions, it is necessary to predict the future point in a specific sequence. We applied our LSTM in both forward and backward directions for considering the future point in time. We also applied bidirectional long short term evaluation of the PHND. We used a one-dimensional bidirectional LSTM (BLSTM) network in our work. The BLSTM is a connectionist classification method which is based on LSTM, RNNs, and bidirectional neural network [55, 56]. The literature reported that BLSTM has much better results for sequence learning.

We selected different parameters of the proposed BLSTM architecture empirically. We fixed the LSTM cells 120 at the hidden layer in our work. Our proposed architecture consists of three layers, i.e., input, hidden, and output layer. As size of the images in our experiments is 48 × 48, the size of the input layer was also 48. The hidden layer has 120 LSTM units, which is followed by the input layer. The output layer, which is also known as the Transaction layer, represents the classification labels in a one-dimensional array, which in our case were 10.

## Standard deep learning models

Existing deep learning models show good performance when these models are used as feature descriptors in various application domains [57]. We use three different SOA deep learning architectures, namely GoogleNet [58], ResNet [59], and AlexNet [60]. We select these models based on their better performance in different application domains. GoogleNet is composed of 22 layers and AlexNet 8 weighted layers. ResNet is available in different configuration, we use 101 layers for ResNet. We extract features from the fully connected layers and did not use any fine tunning.

Table 3 shows summary of the three deep learning architectures which are used for feature extraction and classification. All information about the number of layers, parameters, and feature extraction layer is provided in Table 3. We extract features with these pre-trained models and then train a support vector machine classifier for training. For support vector machine implementation we use Fit multiclass model with default parameters.

**Table 3:** Summary of the standard deep learning architectures parameters: Number of layers, and feature extraction layer for each model used in the proposed work on database PHND.

Method	Number of layers	Number of parameters	Feature extraction layer
GoogleNet [58]	22	102	Cls-FC-2
AlexNet [60]	8	60	FC-7
ResNet [59]	101	44.5	FC-1000

## Experiments and results discussion

### Experimental setup

We performed our experiments with Intel i7 CPU having 16G RAM. The graphical processing unit used was the NVIDIA 840M graphics card. All the tests were performed with Google Tensor-flow and Keras in the Python environment. We trained the model for 30 Epochs while keeping the batch training size as 125. We evaluated our proposed framework on two handwritten digits datasets, including PHDN and CMATERdb 3.1.1. Details about the experimental setup are as follow.

**Fig 4:**
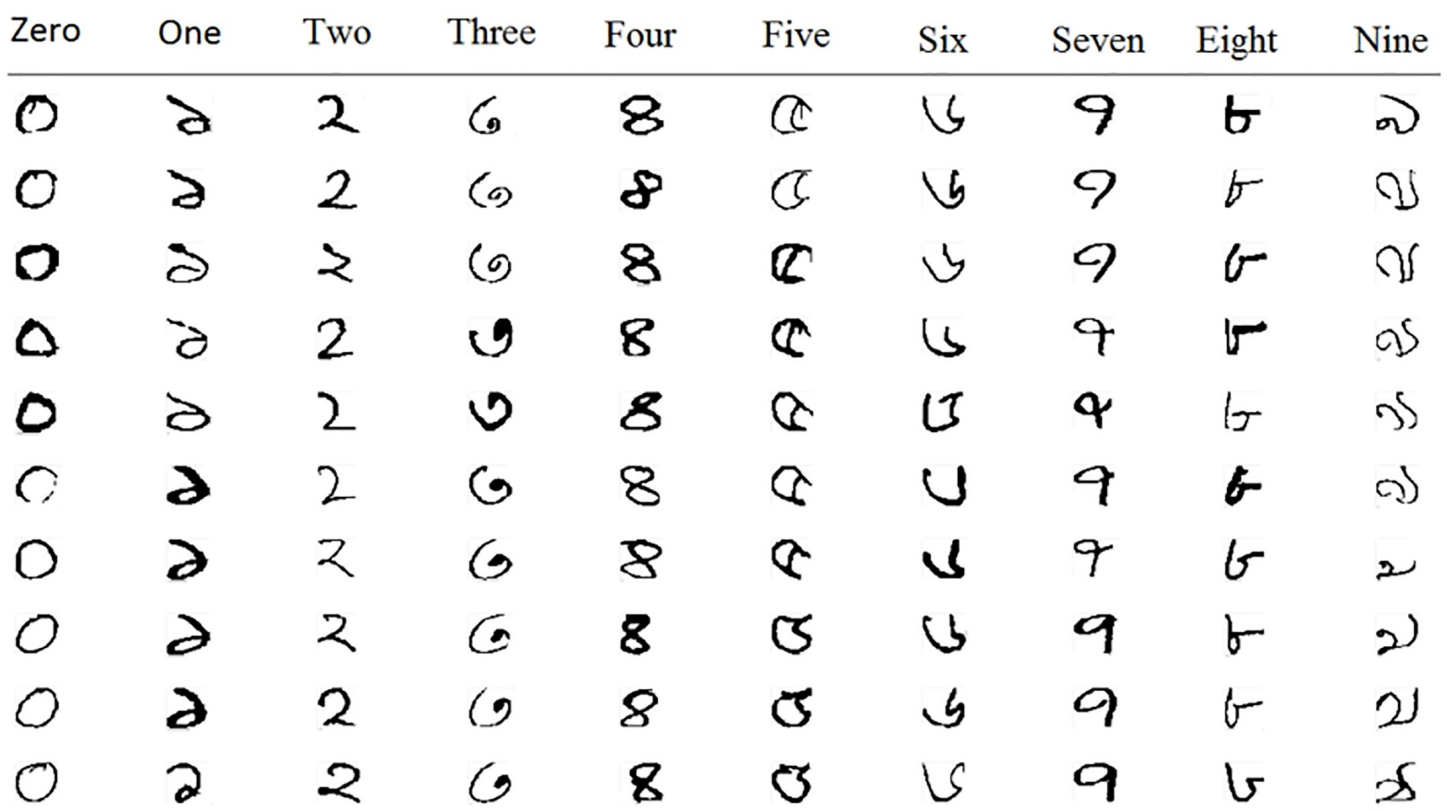
Some sample images from the CMATERdb 3.1.1 database [25].

- The only dataset available for Pashtu handwritten numerals recognition is PHND. We performed the training process with 40,000 samples and testing with the remaining 10,000 images. The participants 1-1000 images were used for training and 1001-1250 for testing purposes. The database is freely available for research purposes.
- As there is no other Pashtu numerals dataset, we evaluated our framework on the Bangla numeral dataset CMATERdb 3.1.1 [61]. The total number of images in the CMATERdb 3.1.1 are 6000. All the images are handwritten isolated Bangla numerals. Some sample images from the CMATERdb 3.1.1 dataset are shown in Fig 4. As can be seen from Fig 4, there is no noise in the images; however, variability in writing style can be found in the images. We used the image setting in our experiments as in [25]. We randomly selected 5000 images for training and 1000 images for testing.
  The total number of images in the CMATERdb 3.1.1 are 6000. All the images are handwritten isolated Bangla numerals. Some sample images from the CMATERdb 3.1.1 dataset are shown in Fig 4. As can be seen from Fig 4, there is no noise in the images; however, variability in writing style can be found in the images. We used the image setting in our experiments as in [25]. We randomly selected 5000 images for training and 1000 images for testing.

### Results discussion

In this section, we investigated the performance of proposed CNNs based numeral recognition system.

**Fig 5:**
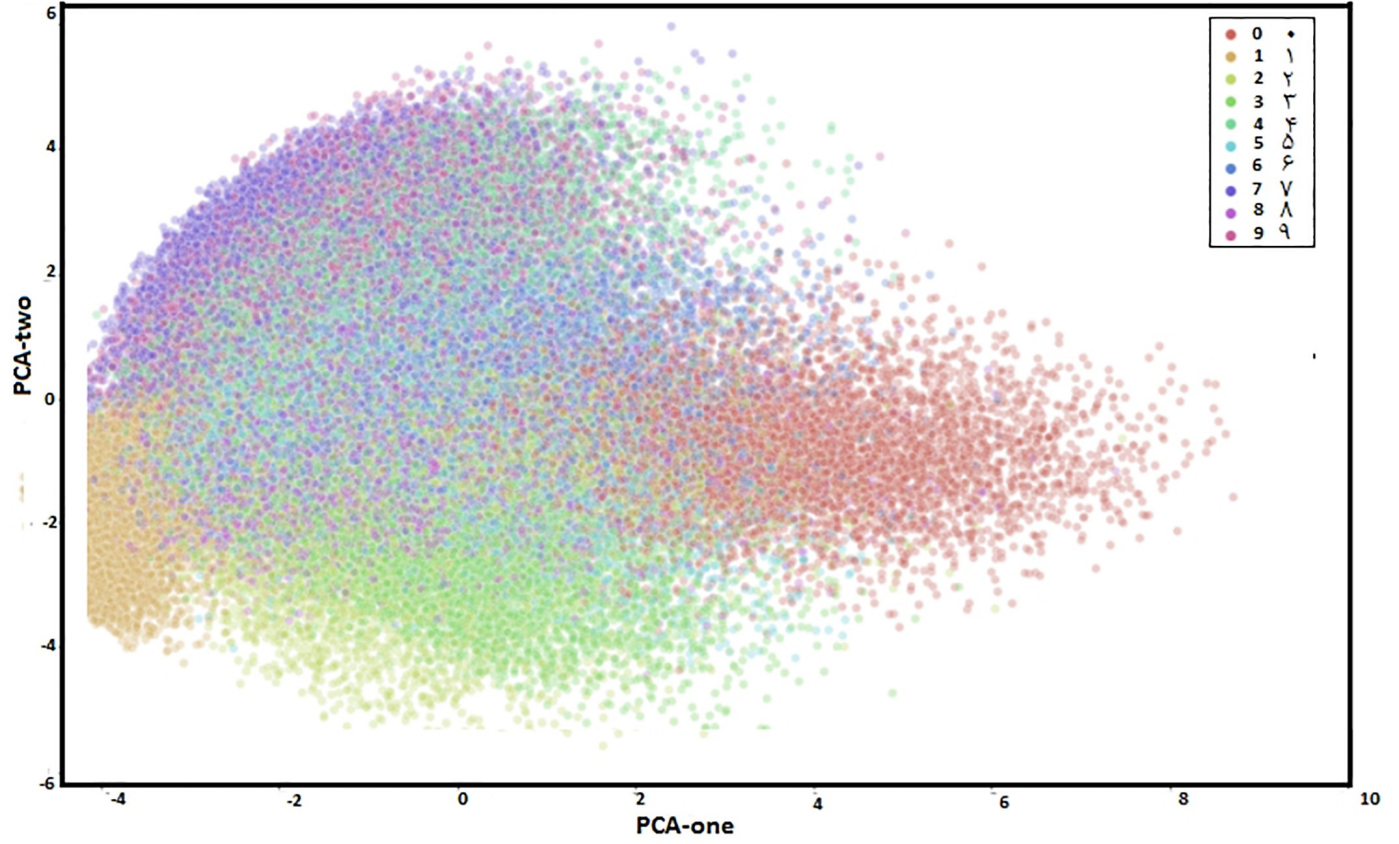
2D visualization of the distribution of data through PCA.

**Fig 6:**
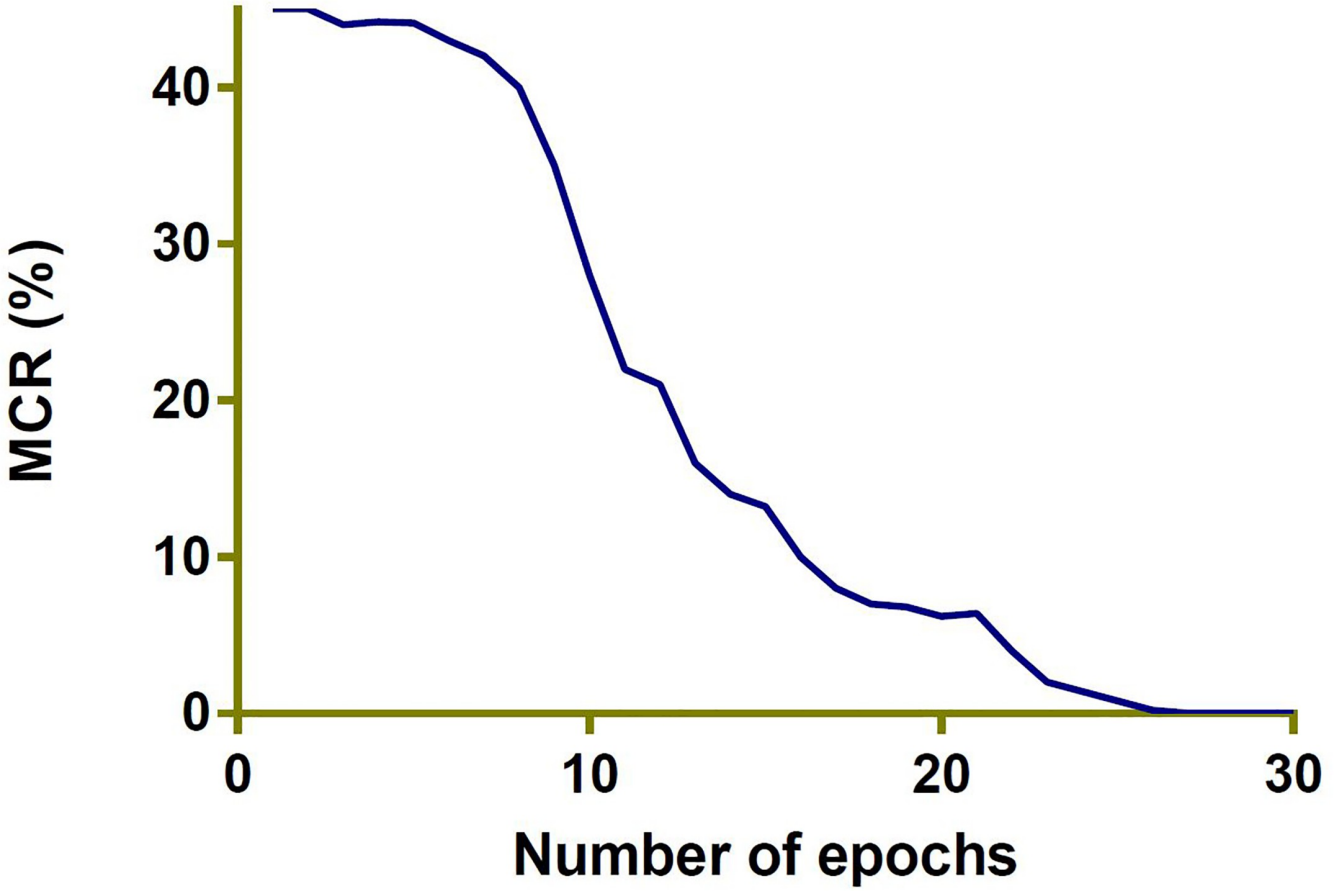
MCR (%) for training data.

- **PHDN**: The only dataset available for Pashtu Numerals is PHND; therefore, we can not compare our results with any other Pashtu Numeral database. Details about the PHND is presented in Section 2. The PHND consists of 50,000 images. We divided the whole dataset into training and testing parts. The training set consists of 4,0000 images, among others. We used participants 1-1000 for the training and remaining 1001 to 1250 for the testing phase.
  Principal Component Analysis (PCA) is a method to reduce the number of dimensions in a particular database while retaining important information. It uses the correlation between some of the total dimensions and also provides fewer variables that keep the maximum amount of information how the original data is distributed. Eigenvalues and eigenvectors of the data matrix is created in this way. The eigenvectors obtained point along the direction of variation in the data. These are the directions with maximum changes in a particular database.
  In Fig 5 scatterplot of the first and second principal components with the color of each digit is shown. The Fig 5 confirms that the two components definitely hold some important information, particularly for specific digits.’
  Fig 6 shows mis-classification rate against the number of epochs. As can be seen the misclassification rate reaches 0 after 25 epochs. As the number of epochs is increased, the classification rate gets improved. For more precise presentation of the results, classification accuracy is shown in the form of confusion matrix in Table 4. From Table 4, it is clear that classification accuracy for some classes (0, 2, 3, 5, and 9) is comparatively lower. The reason is the writing style; for example, 0 is mostly misclassified with the digit 5 due to the similar shape and vice versa. We obtained an average classification accuracy of 98% for PHND. We obtained classification accuracy of 96.3% through DRNNs. We optimized DCNNs in much better way with very detailed experimental work. As a whole, the reported results are encouraging and confirm the effectiveness of the proposed DCNNs and DRNNs frameworks for the task of Pashtu numerals recognition.
  We also evaluate the three standard deep learning models (ResNet, GoogleNet, and AlexNet) in terms of recognition accuracy. Overall we obtained better results with ResNet with 99.1% recognition accuracy. We obtained 98.7% and 98.5% overall recognition accuracy for GoogleNet and AlexNet respectively.
- **CMATERdb 3.1.1**: We noticed much better results for DCNNs as compared to DRNNs with PHND database; hence our experiments with CMATERdb 3.1.1 are limited to DCNNs only. It is a database of Bangla numerals. As no other database is available for Pashtu numerals, we tested our framework on CMATERdb 3.1.1. We use the experimental setup for CMATERdb 3.1.1 as in [25]. We obtained recognition accuracy of 98.64%, which is comparable with SOA results, as shown in Table 5. Accurate comparison on the same set of images as in the methods in Table 5 is not possible, as it is not clear which images were used for training and which for testing. In most of the cases, authors in Table 5 used a random selection of training and testing data.

**Table 4:** Reported confusion matrix for all digits in the database PHND using DCNNs method.

	**Predicted class**										
**True class**		*0*	*1*	*2*	*3*	*4*	*5*	*6*	*7*	*8*	*9*
	*0*	96.43	0.00	0.00	0.00	0.00	3.62	0.00	0.00	0.00	0.00
	*1*	0.00	100	0.00	0.00	0.00	0.00	0.00	0.00	0.00	0.00
	*2*	0.00	0.00	96.82	3.22	0.00	0.00	0.00	0.00	0.00	0.00
	*3*	0.00	0.00	2.48	97.60	0.00	0.00	0.00	0.00	0.00	0.00
	*4*	0.00	0.00	0.00	0.00	100	0.00	0.00	0.00	0.00	0.00
	*5*	6.20	0.00	0.00	0.00	0.00	93.80	0.00	0.00	0.00	0.00
	*6*	0.00	0.00	0.00	0.00	0.00	0.00	98.00	0.00	0.00	2.00
	*7*	0.00	0.00	0.00	0.00	0.00	0.00	0.00	100	0.00	0.00
	*8*	0.00	0.00	0.00	0.00	0.00	0.00	0.00	0.00	100	0.00
	*9*	0.00	0.00	0.00	0.00	0.00	0.00	3.35	0.00	0.00	96.65

**Table 5:** Performance comparison of the proposed method with SOA on CMATERdb 3.1.1 dataset.

Method	Training samples	testing samples	Accuracy (%)
MLP [52]	4000	2000	96.67
MPCA+QTLR [61]	4000	2000	98.55
GA [62]	4000	2000	97.00
Le Net+DBN [25]	5000	1000	98.78
NiN [63]	5000	1000	97.08
Res Net [64]	5000	1000	98.51
Proposed DCNNs based method	5000	1000	98.64
Proposed DRNNs based method	5000	1000	94.64
Fractal Net [65]	4000	2000	98.92
Dense Net [66]	5000	1000	99.13

## Conclusion

This paper introduces a new Pashtu handwritten numerals dataset called PHND. The dataset consists of 50, 000 handwritten images scanned with 300 DPI The database is freely available for downloading and research purposes. A CNNs based method is also introduced for the recognition of the Pashtu digits. The CNNs based model uses two convolutional layers, followed by a Max-Pooling layer. The Re-Lu is used as an activation function in each layer. The FCL layer performs the classification task with Soft-Max. The model is evaluated on two datasets known as PHND and CMATERdb 3.1.1, reporting excellent recognition accuracy. We also perform experiments with RNN and some standard deep learning architectures such as ResNet, GoogleNet, and AlexNet.

Our future work includes an extension of the database to the ligature set. The ligatures will be collected for different fonts of the Pashtu language. After collecting the database, we will move to a complete Pashtu OCR system and its later applications. We have planing to explore both off-line and on-line OCR system for Pashtu language. We intend to target more cursive script languages such as Sindhi, Punjabi, etc. through deep learning-based methods.
